# The Board of Censors, Medical Ethics, Newspapers, Etc.

**Published:** 1897-06

**Authors:** 


### CORRESPONDENCE.

### THE BOARD OF CENSORS, MEDICAL ETHICS, NEWSPAPERS, ETC.

. . . . . . . May 15, 1897.

*Atlanta Medical and Surgical Journal:*

In reviewing the work accomplished by the recent meeting of the Medical Association of Georgia at Macon, the careful, painstaking work of the Board of Censors should receive the approval of the entire profession of Georgia. They acted with proper care in closely investigating every application for membership that came before them, and conscientiously reviewed every complaint or charge brought against the several members of the Association. It is gratifying to these gentlemen, and at the same time is evidence of the thorough good-will of all concerned when it is known that, with only one exception, the gentlemen who went before them came away thoroughly satisfied that not only the character of the profession generally, but their own personal dignity, was maintained by these officers.

This kind of work is not only tedious but very unpleasant. The honor of being a member of the Board of Censors is not sought after, but indeed it is shunned. None of the politicians of the Association ever seek the office for themselves or their friends. As a proof of the confidence of the Association in the gentlemen constituting the board, however, it may be mentioned that one member whose time had expired was unanimously reëlected. These men gave all their time to the duties of the office; one says he did not hear a single paper read, nor know of any of the important transactions of the Association except what occurred in the Board of Censors' room.

During the past year there has been an active effort to enlarge the membership of the Association, particularly in the smaller towns and the rural districts of the State, and an effort has been made to make the Association an aid to the young men of the profession and the country practitioner. This work has been hampered by the complaint that the Association is largely made up of and controled by men from the larger cities who wish to crowd out the younger men and the country doctors. In order to do this, it is claimed they resort to methods which are not in keeping with the dignity of the profession. The newspapers, they say, are filled with pictures of prominent physicians, their private hospitals and patients; accurate accounts of wonderful operations and remarkable cures (couched often in technical terms), and interviews on remarkable cases abroad. The country practitioners claim that this is drawing away from them people who should be their own patients. Whether this be true or not, the Board of Censors determined to make an exhaustive examination of all such charges, not only because such things are unbecoming the dignity of a great profession, but also to protect the young men and the country practitioners in their fields of work.

There are in Georgia numbers of young men well equipped for their duties, ambitious to gain a foothold in the profession by honorable methods, who have accepted the code of ethics as their rule and are living up to it; and there is also a large number of good country practitioners, of riper years, who can and do practice their profession with all the success which their city brothers and the college professors attain. If these men are being injured by methods which, to them, smack of charlatanism and quackery, it is the duty of the Association to do all in its power to protect them, and it would be a brotherly kindness for every prominent member of the profession to guard his name and his successes from the over-ambitious news gatherers.

The practice of medicine is not a trade, the Medical Association of Georgia is not a trades-union, nor is the code of medical ethics a creature of oppression to crush out ambitious men. Medicine is a learned profession, made noble by its object, the relief of suffering and the cure of disease. While it has honored many great men, it has been, *and is now*, honored by men of pure character whose lives were and are above petty self-adulation and struggle for forced growth under the blue glass of small newspaper notoriety.

True, lasting honor will come in the fields of a professsion once honored by One who, while He cured diseases of soul and body, humbly taught the Golden Rule, the code of ethics which is the basis of medical ethics, or any other code under which gentlemen live. This growth will come, with all of its honors, without the aid of sensational notices in the daily press. There is no case in the annals of medical science of a truly great physician who was made great by the secular press, and those who have done the most for our science have least frequently appeared in the public press.

To those who are ambitious to see their names in print and advertise their successes, we would call their attention to one legitimate field for this kind of work—namely, the medical press of the State—here they may honorably disclose this knowledge and give information to those in most need of it and can practically apply it. Confine themselves to this kind of work and they will see fruits that are better and more desirable than those which come from newspaper notoriety. It is a sad thought, however, that we seldom see the same names in the medical journals and the daily paper.

If the Association is to grow in usefulness, it is the duty of every member of it to remove from it any hint that it is a trades-union, organized for the special benefit of the leaders who reside in large cities. An effort during the past few years has been made to eliminate politics from it, and if, as has been suggested, this evil is again creeping in, let us also crush it out as was done in Americus and at other places. All irregularities should be put down, and the officers of the Association should be sustained in their efforts to throw its protection around those most in need of it—the country practitioner and the young doctor.

Following the good work done in Macon, it is sincerely to be regretted that the recent trouble in the local society was allowed to find its way into the secular press of Atlanta. While every one who knows the gentlemen most concerned, believe they are above reproach, it is also true that had the matter been confined to the society it would have been amicably settled by mutual explanations and the gentlemen would have come out firmly in favor of a more vigorous watchfulness over the evils of newspaper interviews. The profession generally has been held up for reproach, and our methods publicly ridiculed. This is unfortunate for our dignity.

The writer of an editorial in a leading sensational sheet of the city certainly must be equally ignorant of both mythological literature and the golden rule which is practiced among gentlemen. He compares the code of medical ethics to the "Celebrated Juggernaut," a heathen god worshiped at Puri, whose devotees believed that it pleased him to have *voluntary* worshipers throw themselves under the wheels of the car containing the idol when it was drawn out at annual festivals. The code of medical ethics is too Christian an institution to insist that any follower of its rule should *voluntarily* sacrifice their dignity, but, perhaps, to please the able editor, we may say any one is at perfect liberty to so do.

Every profession, worthy of that name, may we include journalism, has certain rules by which its members may be measured; it is folly for him, the editor, to try to prejudice the public mind against an instrument which has for its main object the protection of the public against charlatanism and quackery. It is true, as he says, that the code is "ancient," yet it was not "invented in the dark age to be used in medical inquisition," but it is a creation of the good old days when our fathers were true men, its object being to define the position of the honest practitioner, and arose from a need to protect the public *partly* from the public press which was fast degenerating into a hawker of crimes, promoter of political intrigue, and a developer of journalistic quackery, otherwise known at the present time as progressive journalism.

"Its machinery," as the editorial says, "is a little rusty, its cogs creaky, and its steering apparatus out of gear," yet all this comes from the forbearance of a long-suffering profession, which has trusted in the nobility of its followers and attributed much that has passed into the papers, not to members of the profession, but to the hustling newspaper reporter who would turn heaven into hell to get a soul-disgusting article for his *Morning Ventilator* of all that is vile and low.

It is a pitty that the better element of our profession has not yet recognized that the newspaper men themselves, as a retired gentleman of that profession recently told the writer, "regard all this kind of matter as trade-advertisement, and would be surprised if one of our number would come forward and report the deaths occurring in his practice, as it would be *apt to hurt his trade.*" If they did recognize this, we are sure they would shun the reporter as conscientiously as they do open quackery.

We trust that the unkindly criticism to which the profession and its code have been subjected will not interfere with the efforts to purify the profession from all that suggests charlatanism and elevate it above a trade. Let it be known that the same press which would use as space-fillers the name of any doctor or his successful operation, will for the same purpose vilify the profession to which he belongs; self-respect will then demand that to avoid the public press is the highest mark of a gentlemanly dignity. CODE.

